# Interference between the glass, gel, and gas-liquid transitions

**DOI:** 10.1038/s41598-019-52591-x

**Published:** 2019-11-11

**Authors:** José Manuel Olais-Govea, Leticia López-Flores, Jesús Benigno Zepeda-López, Magdaleno Medina-Noyola

**Affiliations:** 1 Instituto de Física “Manuel Sandoval Vallarta”, Universidad Autónoma de San Luis Potosí, Álvaro Obregón 64, 78000 San Luis Potosí, SLP Mexico; 2 Tecnologico de Monterrey, Escuela de Ingeniería y Ciencias, Av. Eugenio Garza Sada 300, 78211 San Luis Potosí, SLP Mexico; 3 Tecnologico de Monterrey, Writing Lab, TecLab, Vicerrectoría de Investigación y Transferencia de Tecnología, Monterrey, 64849 NL Mexico

**Keywords:** Phase transitions and critical phenomena, Condensed-matter physics

## Abstract

Recent experiments and computer simulations have revealed intriguing phenomenological fingerprints of the interference between the ordinary equilibrium gas-liquid phase transition and the non-equilibrium glass and gel transitions. We thus now know, for example, that the liquid-gas spinodal line and the glass transition loci intersect at a finite temperature and density, that when the gel and the glass transitions meet, mechanisms for multistep relaxation emerge, and that the formation of gels exhibits puzzling latency effects. In this work we demonstrate that the kinetic perspective of the non-equilibrium self-consistent generalized Langevin equation (NE-SCGLE) theory of irreversible processes in liquids provides a unifying first-principles microscopic theoretical framework to describe these and other phenomena associated with spinodal decomposition, gelation, glass transition, and their combinations. The resulting scenario is in reality the competition between two kinetically limiting behaviors, associated with the two distinct dynamic arrest transitions in which the liquid-glass line is predicted to bifurcate at low densities, below its intersection with the spinodal line.

## Introduction

Compressing and/or cooling a liquid may lead to its crystalline solidification or to its gas-liquid phase separation^1^. Gases, liquids and (crystalline) solids are equilibrium phases, in principle understood in terms of molecular interactions thanks to Boltzmann’s fundamental expression for the entropy, *S* = *k*_*B*_ ln*W*, and to the maximum-entropy principle^2^. A simple and intuitive use of these principles is illustrated by van der Waals explanation of the origin of gases and liquids and their coexistence^3,4^. In practice, however, compressing and/or cooling a liquid may also lead to the formation of compact and relatively homogeneous amorphous solids (glasses) and of more tenuous sponge-like amorphous materials (gels), that pervade our daily life^5–7^.

So far, however, we do not know how Boltzmann’s principle operates to govern the formation of these non-equilibrium amorphous solids that do not maximize entropy. Such fundamental knowledge would allow us to systematically build an understanding (even at “van der Waals” level) of the exploding experimental and computational information^8,9^ on the general phenomenology of these non-equilibrium phases. One of the most illustrative examples is the amazing behavior observed when the ordinary equilibrium gas-liquid phase transition interferes with the non-equilibrium glass transition^10^, leading to remarkable multistep relaxation processes^11–13^ and puzzling delay (or “latency”) effects^14–16^ during the formation of gels by arrested spinodal decomposition.

The main aim of this work is to demonstrate that a microscopic statistical mechanical framework to understand this interplay between gas-liquid spinodal decomposition, gelation and the glass transition, results from a straightforward application of the non-equilibrium self-consistent generalized Langevin equation (NE-SCGLE) theory of irreversible processes in liquids^17,18^ to a Lennard-Jones–like (“LJ-like”) simple liquid (pair interaction = harsh repulsion + longer-ranged attraction).

The NE-SCGLE theory originates from a generalization of Onsager’s description of irreversible processes and fluctuations, to genuine non-equilibrium and non-linear conditions. Applied to the description of irreversible processes in liquids, this canonical and abstract formalism becomes a generic first-principles theory of its structure and dynamics, at equilibrium and during the non-stationary processes of equilibration or aging. In the Supplemental Material (SM) we briefly review the genesis and achievements of this non-equilibrium theory, and guide the reader through the pertinent references. There we also write in detail the self-consistent system of equations (Eqs (SM6)–(SM11)) that summarizes the simplest approximate version of the NE-SCGLE theory employed in this and in previous studies^18–22^.

As recently shown^19^, the transformation of equilibrium hard-sphere (and soft-sphere) liquids into “repulsive” glasses, provided by the solution of these NE-SCGLE equations, naturally explains some of the most essential nonequilibrium signatures of the glass transition (such as the aging of the structure and dynamics)^5–7^. When applied to LJ-like simple liquids, the same NE-SCGLE equations predict new dynamically-arrested phases, identified with gels and porous glasses^20^, and provides a kinetic perspective of the irreversible evolution of the structure of the system after being instantaneously quenched to the interior of its spinodal region^21,22^. The main aim of the following discussion is to illustrate the NE-SCGLE prediction that the seemingly complex interplay between spinodal decomposition, gelation, glass transition, and their combinations^11–13^, may be simply understood in terms of two kinetically competing limiting behaviors, associated with the two underlying dynamic arrest transitions predicted to exist^20^ in the gas-liquid spinodal region by the very same NE-SCGLE Equation.

## Results and Discussion

As in refs.^20–22^, here we also refer for concreteness to a representative Brownian LJ–like model system, namely, the “hard-sphere plus attractive Yukawa” (HSAY) potential

1 $$u(r)=\{\begin{array}{lc}\infty , & r < \sigma ;\\ -\epsilon \frac{\exp [\,-\,z(r/\sigma -\mathrm{1)]}}{(r/\sigma )}, & r > \sigma ,\end{array}$$

whose state space is spanned by the number density *n* and the temperature *T*, expressed in dimensionless form as [*nσ*^3^] and [*k*_*B*_*T*/$\epsilon$] (with *k*_*B*_ being Boltzmann’s constant), and denoted simply as *n* and *T*. We shall also refer to the hard-sphere volume fraction *ϕ* ≡ *πn*/6. Complementing the recent study of the evolution of the non-equilibrium structure factor *S*(*k*; *t*_*w*_)^21^, in this paper we shall discuss the full solution of the referred NE-SCGLE equations at *all* waiting times *t*_*w*_, but focusing now on the kinetics (i.e., the aging) of the non-equilibrium *dynamic* properties.

Let us assume that this system, initially in equilibrium at the state point (*n*, *T*_*i*_), is *instantaneously* quenched at time *t*_*w*_ = 0 to a final temperature *T*_*f*_ under *isochoric* conditions and in the absence of applied external fields. Then the NE-SCGLE description of the irreversible evolution of *S*(*k*; *t*_*w*_) for waiting times *t*_*w*_ > 0 is provided^20^ by the solution of Eq. SM6, i.e.,

2 $$\frac{\partial S(k;{t}_{w})}{\partial {t}_{w}}=-\,2{k}^{2}{D}^{0}b({t}_{w})n{ {\mathcal E} }_{f}(k)[S(k;{t}_{w})-1/n{ {\mathcal E} }_{f}(k)]\mathrm{.}$$

In this equation *D*^0^ is the short-time self-diffusion coefficient (see the SM) and ${ {\mathcal E} }_{f}(k)$ is the Fourier transform (FT) of ${\mathcal E} (r;n,{T}_{f})$, which is the functional derivative ${\mathcal E} [|r-r^{\prime} |;n,T]\equiv [\delta \beta \mu [r;n,T]/\delta n(r^{\prime} )]$ of the local chemical potential (in units of the thermal energy *k*_*B*_*T* ≡ 1/*β*), evaluated at the uniform density and temperature profiles *n*(*r*) = *n* and *T*(*r*) = *T*_*f*_. As in refs^20,21^, ${ {\mathcal E} }_{f}(k)$ will be approximated by its exact hard-sphere value ${ {\mathcal E} }_{HS}(k;\varphi )$ plus the FT of the attractive Yukawa tail, ${ {\mathcal E} }_{f}(k;n,{T}_{f})={ {\mathcal E} }_{HS}(k;\varphi )+\beta {u}_{A}(k)$.

**Figure 1:**
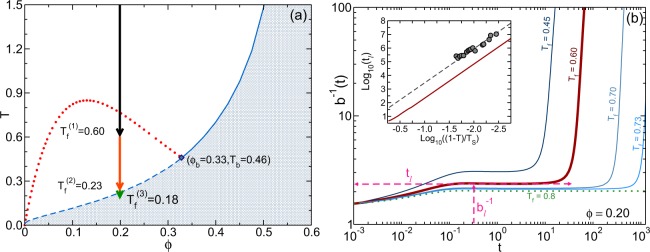
(**a**) Dynamic arrest transitions of the HSAY liquid (*z* = 2) from ref.^20^: liquid-glass (solid line) and gel-glass (dashed line) transitions, and “type A” dynamic arrest transition (dotted line), which coincides with the spinodal line *T* = *T*_*s*_(*ϕ*) to the left of the bifurcation point (solid diamond). Downward arrows 1–3 represent instantaneous temperature quenches discussed in the text. (**b**) Thick solid line: waiting-time dependence of the (inverse) mobility *b*(*t*) for quench 1 (final temperature *T*_*f*_^(1)^ = 0.6), exhibiting the plateau *b*_*l*_(*T*_*f*_) lasting a latency time *t*_*l*_(*T*_*f*_). The other lines correspond to other values of *T*_*f*_ in the neighborhood but below (softer solid lines) and above (dashed line) the spinodal. The inset compares the predicted divergence *t*_*l*_(*T*) ∝ (1 − *T*/*T*_*s*_)^−2.5^ (solid line) with the experimental data of ref.^14^ (circles), fitted by the same power law (dashed line).

The dimensionless mobility function *b*(*t*_*w*_) in Eq. 2 is in reality a state function, determined by the full set of NE-SCGLE equations, constituted by Eq. (2) itself, together with Eqs. (SM7)-(SM11). From the analysis of the long-time asymptotic solutions of these equations, it was shown in ref.^20^. that the liquid-glass transition of Lennard-Jones–like liquids (solid line of Fig. 1(a) for the HSAY model) indeed intersects the spinodal line, as previously discovered by simulations^10^. The NE-SCGLE theory, however, goes beyond, to predict in addition that below the intersection point (*ϕ*_*b*_, *T*_*b*_), the liquid-glass transition bifurcates in two dynamic arrest transitions (dotted and dashed lines of Fig. 1(a), explained in detail in ref.^20^). In what follows we shall demonstrate that, in addition, the other observed fingerprints of the interference between the glass and the gas-liquid transitions are also part of the unified and detailed scenario predicted by the NE-SCGLE theory.

The three downward arrows in Fig. 1(a) represent instantaneous temperature quenches along the isochore *ϕ* = 0.2 from a common initial temperature *T*_*i*_ = 1.5, to the indicated lower final temperature *T*_*f*_. Quench 1 represents the regime of shallow quenches. Its final temperature *T*_*f*_^(1)^ = 0.6 lies near but *below* the first non-equilibrium transition (dotted line). This “type-A” transition^20^ falls on top of the gas-liquid spinodal curve *T* = *T*_*s*_(*ϕ*), and for this isochore, *T*_*s*_(*ϕ* = 0.2) = 0.764. Quenches 2 and 3 are representative of the regime of deep quenches, where *T*_*f*_ lies in the neighborhood of the lower-temperature (“type-B”) gel-glass transition *T* = *T*_*c*_(*ϕ*)^20^ (dashed line). This is clearly just the continuation inside the spinodal region, of the “ordinary” liquid-glass transition (solid line of Fig. 1(a)).

The irreversible structural relaxation that follows these quenches manifests itself most dramatically, and with rather unexpected consequences, in the evolution of the non-equilibrium dynamics, which exhibits a complex time-temperature-density dependence, as illustrated and summarized by the mobility *b*(*t*_*w*_). For example, the thick solid line of Fig. 1(b), labeled *T*_*f*_ = 0.6, corresponds to quench 1, and illustrates the most salient kinetic feature of shallow quenches: the inverse *b*^−1^(*t*_*w*_), expected to mimic the structural relaxation time or the viscosity, exhibits a remarkable “latency” period, of duration *t*_*l*_, in which it reaches a pseudo-equilibration plateau with constant value *b*_*l*_^−1^. Within this latency time, the particles are able to diffuse a distance ${d}_{l}\equiv \sqrt{{b}_{l}{t}_{l}/6}$. At the end of this latency period, *b*^−1^(*t*_*w*_) diverges with *t*_*w*_ as dynamic arrest now fully develops.

As illustrated by the other (thinner) solid lines of Fig. 1(b) (which represent other shallow quenches), *t*_*l*_, *b*_*l*_ and *d*_*l*_ depend on the final temperature *T*_*f*_ of the quench, increasing monotonically when *T*_*f*_ approaches *T*_*s*_ from below. A more detailed calculation, illustrated in the inset, reveals that the predicted *t*_*l*_(*T*_*f*_) actually diverges at the spinodal temperature *T*_*s*_ as *t*_*l*_ ∝ (1 − *T*_*f*_/*T*_*s*_)^−*α*^, with an exponent *α* ≈ 2.5. It is natural to expect that this kinetic behavior of *b*^−1^(*t*_*w*_) will also be observed more directly in the aging kinetics of the viscosity and of the *α*-relaxation time. In fact, it is quite remarkable that this predicted latency effect has actually been experimentally observed in rheometry experiments during gel formation in weakly attractive nanocolloid suspensions^14^. Thus, for quenches along the isochore *ϕ* = 0.2, the *experimental* latency time, denoted in^14^ as *t*_*G*_(*T*_*f*_), was observed to diverge as *t*_*G*_ ∝ (1 − *T*_*f*_/T^*^)^−*α*^ with *α* ≈ 2.5 (see Fig. 7(a) of^14^). The agreement of our theoretical prediction with these results, presented in the inset of Fig. 1(b), and discussed in more detail in the Supplemental Material, allows us to identify the singular temperature T^*^ empirically determined in ref.^14^, with the spinodal temperature *T*_*s*_.

**Figure 2:**
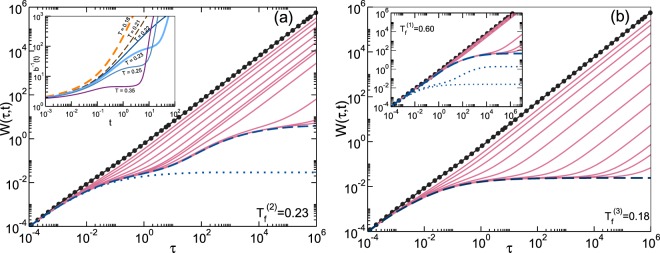
Mean square displacement *W*(*τ*; *t*_*w*_, *T*_*f*_) as a function of correlation time *τ* for waiting times *t*_*w*_ = 0 (line with dark circles), *t*_*w*_ = 10^0.5*n*^, with *n* = −3, −2, −1, … (solid lines), and *t*_*w*_ = ∞ (dashed lines) after a quench to final temperature *T*_*f*_^(2)^ = 0.23 (main panel (a)), *T*_*f*_^(3)^ = 0.18 (main panel (b)) and *T*_*f*_^(1)^ = 0.60 (inset (b)). For reference we also include the asymptotic curves *W*_*a*_(*τ*; *T*_*f*_) ≡ *W*(*τ*; *t*_*w*_ = ∞, *T*_*f*_) (dotted lines) for *T*_*f*_ = 0.18 in (**a**) and *T*_*f*_ = 0.18 and 0.23 in the inset of (**b**). The inset of (**a**) contrasts the behavior of (the inverse of) *b*(*t*; *T*_*f*_) for *T*_*f*_ slightly above (solid lines) and below (dashed lines) the gel-glass transition line.

In contrast to approaching the spinodal line from below, let us now go in the opposite direction (i.e., decreasing *T*_*f*_ toward *T*_*c*_). We then observe that *t*_*l*_(*T*_*f*_) and *b*_*l*_(*T*_*f*_) decrease with *T*_*f*_ until the latency plateau transforms into a mild and fading inflection point. This is illustrated by the solid curves in the inset of Fig. 2(a). The dashed lines correspond to final temperatures *below* the gel-glass transition *T* = *T*_*c*_(*ϕ*), and serve to illustrate the complete absence of latency effects below *T*_*c*_. In fact, the main feature to highlight in Fig. 2 is precisely this striking kinetic difference, predicted to occur when *T*_*f*_ crosses the gel-glass transition line. This is illustrated by quenches 2 and 3, chosen such that *T*_*f*_ lies, respectively, slightly above (*T*_*f*_^(2)^ = 0.23) and slightly below (*T*_*f*_^(3)^ = 0.18) this transition (which occurs at *T*_*c*_(*ϕ* = 0.2) = 0.22). In Fig. 2(a,b) this kinetic difference is exhibited in more detail in terms of the different pattern of aging of the mean squared displacement *W*(*τ*; *t*_*w*_, *T*_*f*_) ≡ <[R(*t*_*w*_ + *τ*) − R(*t*_*w*_)]^2^>/6*σ*^2^ of these two quenches, plotted as a function of the correlation time *τ* (solid lines), for the same sequence of values of the waiting time *t*_*w*_ (equally-spaced in log *t*_*w*_).

As observed in Fig. 2(a), quench 2 presents a remnant of latency effects, observed as the inflection point in the *t*_*w*_-dependence of *b*^−1^(*t*_*w*_) in the inset of Fig. 2(a) (thicker solid line), and in the crowding of the solid lines for *W*(*τ*; *t*_*w*_, *T*_*f*_^(2)^) corresponding to ${t}_{w}\lesssim {t}_{l}$, relative to the kinetics of quench 3, presented in Fig. 2(b). The latter is pretty similar to the more uniform and faster aging pattern predicted and observed in the formation of repulsive glasses (see, e.g., Fig. 15 of^23^). Such pattern involves an asymptotic localization length *λ*_*HS*_ of about 0.14 *σ*, a value reminiscent of Lindemann’s criterion of melting, suggestive of a hard-sphere caging mechanism of arrest^24^. This is consistent with the formation of compact, but porous, repulsive glasses. In fact, a localization length *λ*_*HS*_ ≈ 0.14*σ* is predicted all along the gel-glass and liquid-glass portions of the glass transition line, up to the hard-sphere glass-transition at (*ϕ* = 0.582, *T* = ∞).

The other most notorious fingerprint of the interference between gelation and glass transition, predicted by the NE-SCGLE theory, is also illustrated by quench 2 in Fig. 2(a). This is in reality another manifestation of the inflection point exhibited by *b*^−1^(*t*_*w*_) for *T*_*f*_ slightly above *T*_*c*_. We refer to the build-up, at long waiting times, of a two-step long-*τ* relaxation of the main dynamic properties. Notice, for example, that for waiting times smaller than the latency time (${t}_{w}\lesssim {t}_{l}$), *W*(*τ*; *t*_*w*_, *T*_*f*_^(2)^) bends towards the asymptotic MSD *W*_*a*_(*τ*; *T*^(3)^) ≡ *W*(*τ*; *t*_*w*_ = ∞,*T*_*f*_^(3)^) of quench 3 (dotted line, with localization length *λ*_*HS*_ ≈ 0.14*σ*), thus suggesting an initial tendency to arrest through a hard-sphere caging mechanism.

At waiting times *t*_*w*_ much longer than the latency time *t*_*l*_, however, this behavior is “corrected”, now bending toward the “true” predicted asymptotic MSD *W*_*a*_(*τ*; *T*^(2)^) ≡ *W*(*τ*; *t*_*w*_ = ∞, *T*_*f*_^(2)^) of quench 2 (dashed line), whose localization length *λ*_*gel*_ is a few times larger, reflecting the localization of the particles within the transient particle network of the gel phase^12^. This is highly reminiscent of the early proposal^25^ that colloid gelation is the result of a two-level dynamical arrest process, first at the level of the colloidal particles leading to clusters, and then at the level of clusters undergoing a glass transition. This notion, first formalized using mode coupling theory^26^, and later framed^27^ in terms of the Cauchy-Born theory of amorphous solids^28^, has been recently confirmed by experimental observations using different techniques^29^. The present NE-SCGLE approach, whose equilibrium version is conceptually closer to MCT (see a recent and detailed discussion in ref.^30^), provides a complementary kinetic perspective, predicting the two-stage aging of the dynamics without assuming a priori a two-level arrest scenario.

It is important to mention that this complex pattern of aging has been observed in the simulation of the non-equilibrium dynamics of suspensions of HS-like particles transiently bonded by cross-linking polymeric agents, whose net effect is an effective attraction between the particles (see, e.g., Fig. 4 of^11^ and Fig. 11 of^13^). Although not shown here (see, however, the SM), the corresponding two-step relaxation is also exhibited by the self-intermediate scattering function (self-ISF) *F*_*S*_(*k*, *τ*; *t*_*w*_) ≡ <exp *i*k · [R(*t*_*w*_ + *τ*) − R(*t*_*w*_)]>.

The main features of the NE-SCGLE predicted scenario of the interference between the gas-liquid and the glass transitions may thus be summarized by the conclusion that the latency time and the double relaxation are in reality the most representative manifestations of two competing and complementary extreme behaviors. The first of them was discussed in Fig. 1(b), and refers to the appearance of latency effects associated with the dynamic arrest transition line *T* = *T*_*s*_(*ϕ*) (at which the latency time *t*_*l*_(*T*_*f*_) diverges). Thus, while for *T*_*f*_ above *T*_*s*_ the system will always reach a homogeneous equilibrium state, for *T*_*f*_ below *T*_*s*_ no homogeneous equilibration is possible. Instead, for *T*_*f*_ below but sufficiently close to *T*_*s*_, no signs of arrest may be observed, due to the very long latency time *t*_*l*_(*T*_*f*_), thus allowing the system to undergo full *inhomogeneous* gas-liquid equilibrium phase separation.

Notice in the inset of Fig. 2(b) that in this regime of shallow quenches, the two-step relaxation is virtually absent. The occurrence of inhomogeneous phase separation, however, renders this observation rather irrelevant in practice. Nevertheless, it is still interesting to notice that the divergence of *t*_*l*_(*T*_*f*_) and *d*_*l*_(*T*_*f*_) when *T*_*f*_ approaches *T*_*s*_ from below, is a non-equilibrium dynamic counterpart of the diverging equilibrium correlation length^31^ expected when we approach the critical point (or any point along the spinodal curve) from above. Both originate in the thermodynamic instability represented by the spinodal line, information that enters in the NE-SCGLE theory through the thermodynamic input ${\mathcal E} (k;n,{T}_{f})$.

The second complementary extreme behavior is associated with crossing the gel-glass transition line *T* = *T*_*c*_(*ϕ*). Thus, very deep quenches (*T*_*f*_ below *T*_*c*_, illustrated by quench 3) lead to the formation of porous structures made of compact “repulsive” glasses (localization length *λ*_*HS*_ ≈ 0.14*σ*). In contrast, slightly shallower quenches (*T*_*f*_ immediately above *T*_*c*_, quench 2), are predicted to form “fluffier” and less rigid (i.e., more viscoelastic) and highly heterogeneous materials, with localization lengths *λ*_*gel*_ several times larger than *λ*_*HS*_. The kinetic and dynamical fingerprint of these materials, which we identify with gels, is the two-step pattern of structural relaxation illustrated in Fig. 2(a) by quench 2.

According to the predicted scenario, quenches whose *T*_*f*_ lies midway between these two extreme regimes, will involve kinetic processes featuring a combination of these two limiting tendencies. However, since near the spinodal line the unrestricted tendency to gas-liquid phase separation is expected to prevail over the predicted non-equilibrium divergence of *t*_*l*_(*T*_*f*_) and *d*_*l*_(*T*_*f*_), we must conclude that some form of smooth crossover, from full gas-liquid separation to the formation of gels, must occur somewhere between *T*_*s*_ and *T*_*c*_. Although this would then imply that there is nothing like a sharp *gel line*, we might determine the “epicenter” *T*_0_(*ϕ*) of this postulated crossover, as indicative of the rather diffuse boundary between heterogeneous gas-liquid separation and gel formation.

**Figure 3:**
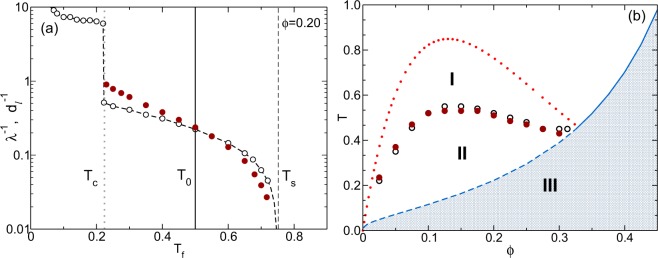
(**a**) Numerical results for the inverse localization length *λ*^−1^(*T*_*f*_) (empty circles) and the inverse latency distance *d*_*l*_^−1^(*T*_*f*_) (solid circles) as a function of *T*_*f*_, along the isochore *ϕ* = 0.2. As discussed in ref.^20^, the crossover from exponential to power-law divergence of *λ*^−1^(*T*_*f*_) occurs at *T*_0_(*ϕ*) (vertical solid line). As explained in the SM, the same procedure can be applied to *d*_*l*_^−1^(*T*_*f*_), yielding *ν* = 1.25, and an alternative determination of *T*_0_(*ϕ*). (**b**) Crossover temperature *T*_0_(*ϕ*) obtained from *λ*(*T*_*f*_) (empty circles) and from *d*_*l*_(*T*_*f*_) (solid circles), represented on the non-Equilibrium phase diagram of Fig. 1(a). Regions I, II, and III correspond, respectively, to full gas-liquid phase separation, to gel formation, and to the formation of porous glasses.

In ref.^20^ an empirical criterion was suggested to determine *T*_0_(*ϕ*), based on the *T*_*f*_-dependence of the infinitely “aged” (*t*_*w*_ → ∞) mean square displacement, *W*_*a*_(*τ*; *T*_*f*_) ≡ *W*(*τ*; *t*_*w*_ = ∞, *T*_*f*_) (illustrated by the dotted and dashed lines in Fig. 2(a,b)). The arrested plateau *W*_*a*_(*τ* → ∞, *T*_*f*_) defines the long-time asymptotic value of the squared localization length, *λ*^2^(*T*_*f*_) ≡ *W*_*a*_(*τ* → ∞, *T*_*f*_), whose *T*_*f*_-dependence is illustrated in Fig. 3(a) for the isochore *ϕ* = 0.2. For *T*_*f*_ above but near *T*_*c*_, *λ*(*T*_*f*_) increases exponentially with *T*_*f*_, whereas for *T*_*f*_ below but near *T*_*s*_, *λ*(*T*_*f*_) diverges as (1 − *T*_*f*_/*T*_*s*_)^−*ν*^ with *ν* = 0.75. As explained in ref.^20^. (where *λ*^2^(*T*_*f*_) is denoted as *γ*_*a*_(*T*_*f*_)), this allows us to determine a crossover temperature *T*_0_ between these two regimes. Applying this procedure at other isochores led to the determination of *T*_0_(*ϕ*) reported in Fig. 10 of ref.^20^, and reproduced here as the empty circles of Fig. 3(a).

Let us now mention that the same notion, derived in ref.^20^ from long-time asymptotic properties, also emerges from the *T*_*f*_-dependence of finite-waiting-time properties, more specifically, of the latency distance *d*_*l*_(*T*_*f*_). This provides us with a similar but independent determination of *T*_0_(*ϕ*). To see this, in Fig. 3(a) we present the predicted *d*_*l*_(*T*_*f*_) as a function of *T*_*f*_ along the isochore *ϕ* = 0.2. As explained in detail in the SM, the *T*_*f*_-dependence of *d*_*l*_(*T*_*f*_) also happens to exhibit an exponential to power-law crossover, and hence, we can also determine a corresponding crossover temperature *T*_0_(*ϕ*). The result of this procedure are illustrated by the solid circles of Fig. 3(b).

Clearly, both routes determine essentially the same location of the crossover temperature *T*_0_(*ϕ*). Although we have not demonstrated that these points correspond to the gel line, this coincidence is reassuring, and we may take them as indicative of the diffuse boundary, above which the system is more likely to phase separate, and below which it is more likely to form a gel. With this provision, the coincidence of both methods to determine *T*_0_(*ϕ*), lends additional support to the scenario proposed in ref.^20^, that regions I (*T*_0_(*ϕ*) < *T*_*f*_ < *T*_*s*_(*ϕ*)), II (*T*_*c*_(*ϕ*) < *T*_*f*_ < *T*_0_(*ϕ*)), and III (*T*_*f*_ < *T*_*c*_(*ϕ*)) correspond, respectively, to full gas-liquid phase separation, to gel formation, and to the formation of porous glasses. This predicted non-equilibrium phase diagram (NEPD) is strongly reminiscent of the experimentally-determined NEPD reported in Fig. 4 of ref.^32^ and of the theoretically-proposed NEPD presented in Fig. 3 of ref.^27^.

## Conclusion

Let us conclude with a word of cautious optimism. The scenario presented here and in refs^20–22^ illustrates the general features predicted by the NE-SCGLE theory regarding the non-equilibrium structural and dynamical evolution leading to the formation of arrested states after quenching a LJ-like simple liquid inside its spinodal region. Some detailed features, however, may depend on the detailed conditions, whose discussion was left out of the scope of this manuscript. We refer, for example, to the variation of the form and range of the pair potential and of the specific thermal manipulation protocol (beyond the most primitive one considered in this work, namely, the instantaneous homogeneous temperature quench).

The good news is that there seems to be no fundamental obstacles to incorporate these effects in additional applications of the NE-SCGLE theory, as we expect to illustrate in separate communications. For the time being, we may conclude that the qualitative scenario provided by the non-equilibrium SCGLE theory, within its simplest version and assumptions, seems to provide a sound and illuminating perspective to the understanding of the interference between one equilibrium phase transition (the gas-liquid phase separation in the present case) and a non-equilibrium kinetic arrest transition (represented here by the glass transition).

## Supplementary information

Supplementary material
