# Supplementary material for: Interference between the glass, gel, and gas-liquid transitions
Source: Sci Rep. 2019 Nov 11;9:16445. doi: 10.1038/s41598-019-52591-x (PMC6848111; doi:10.1038/s41598-019-52591-x)
Supplement: Supplementary file 1 — Supplementary material [file 41598_2019_52591_MOESM1_ESM.pdf]

How the glass, gel, and gas-liquid transitions interfere. Supplemental Material

José Manuel Olais-Govea[1,2], Leticia López-Flores[1*], Benigno Zepeda-López[1], and Magdaleno Medina-Noyola[1]
[1] *Instituto de Física "Manuel Sandoval Vallarta", Universidad Autónoma de San Luis Potosí, Álvaro Obregón 64, 78000 San Luis Potosí, SLP, México.*
[2] *Tecnologico de Monterrey, Escuela de Ingeniería y Ciencias, Av. Eugenio Garza Sada 300, 78211, San Luis Potosí, SLP, México.*
[3] *Tecnologico de Monterrey, WritingLab, TecLab, Vicerrectoría de Investigación y Transferencia de Tecnología, Monterrey 64849, NL, Mexico.*

THE NE-SCGLE THEORY.

This section is a brief review through the pertinent references describing the antecedents and origin of the *non-equilibrium* self-consistent generalized Langevin equation (NE-SCGLE) theory of irreversible processes in liquids [1]. Here we highlight the main simplifying approximations leading to the lowest-order version of the NE-SCGLE theory (Eqs (SM6)-(SM11) below), employed in practice in this and in all previous studies [2, 3].

Origin of the NE-SCGLE theory.

Mode coupling theory (MCT) [4–7] stands as the first and best established *microscopic* theory of the glass transition, given its many experimentally confirmed predictions [8, 9]. More recently, the self-consistent generalized Langevin equation (SCGLE) theory [10–14], originally proposed as a theory of colloid dynamics, also became a theory of dynamic arrest [15–21]. Although essentially both theories only describe the dynamics of equilibrium liquids, they can predict the circumstances in which dynamic arrest sets in. Except in some special conditions, both lead in general to similar predicted scenarios, as recently analyzed in all detail [22].

The main reason for this coincidence is that both, MCT and the SCGLE, start from the same fundamental basis, namely, the most general and exact stochastic diffusion equation for the fluctuations of the local density of particles, later complemented by specific simplifying approximations. In MCT, the exact stochastic diffusion equation is derived from a Hamiltonian level of description using Mori-Zwanzig's *equilibrium* projection operator methods [23–26]. In contrast, in the SCGLE theory, the same equation derives from a mathematical model referred to as the *generalized Langevin equation* (GLE). This is defined as the most general *stationary* stochastic process generated by a linear stochastic differential equation with additive noise [27, 28].

Let us represent such "most general *stationary* stochastic process" as the vector $\mathbf{a}(t) = [a_1(t), a_2(t), ..., a_\nu(t)]^\dagger$ (where † means transpose), grouping the set of ν stochastic variables $a_i(t)$ $(i = 1, 2, ..., \nu)$. Then, as explained in Ref. [27], the mathematical attribute of stationarity is a necessary and sufficient condition for the dynamics of the fluctuations $\delta a_i(t)$ $(\equiv a_i(t) - \overline{a}_i^{\,ss})$ around the stationary mean value $\overline{a}_i^{\,ss}$ to be described by the following stochastic differential equation,

$$\frac{d\delta \mathbf{a}(t)}{dt} = -\omega\chi^{-1}\delta\mathbf{a}(t) - \int_0^t L(t-t')\chi^{-1}\delta\mathbf{a}(t')dt' + \mathbf{f}(t). \tag{SM1}$$

In this equation χ is the matrix of static correlations, $\chi_{ij} \equiv \langle \delta a_i(0)\delta a_j^*(0)\rangle$, ω is an anti-Hermitian matrix, $\omega_{ij} = -\omega_{ji}^* \equiv \langle \delta \dot{a}_i(0)\delta a_j^*(0)\rangle$ (with $\langle ...\rangle$ denoting the average over the initial values $\delta\mathbf{a}(0)$), and the memory function matrix $L(t)$ is determined by the fluctuation-dissipation relation $L_{ij}(t) = \overline{f_i(t) f_j^*(0)}$, where $f_i(t)$ is the ith component of the random vector $\mathbf{f}(t)$ and the overline denotes the average over the realizations of $\mathbf{f}(t)$.

Eq. (SM1) is referred to as the *generalized Langevin equation* (GLE). As a mathematical model, it can be freely applied to any (not necessarily physical) *stationary* phenomenon described by a linear equation with additive noise. Thus, no wonder that the thermal fluctuations around the thermodynamic equilibrium state are governed by time-evolution equations with the mathematical structure of Eq. (SM1), with the stationary mean values $\overline{a}_i^{\,ss}$ and covariance matrix χ given by their *equilibrium* value $\overline{a}_i^{\,eq}$ and $\chi^{\,eq}$.

In fact, the GLE in Eq. (SM1) was widely used in the early description of thermal fluctuations in simple liquids [29, 30], although in strong association with its derivation from a hamiltonian level of description through the use of Mori-Zwanzig's *equilibrium* projection operator methods [23–26]. Unfortunately, the mechanistic nature of Mori-Zwanzig's derivation obscures the fact that in reality the mathematical structure of Eq. (SM1) is a consequence of the mathematical condition of stationarity, and not a consequence of such hamiltonian origin. For the same reason, its validity is not restricted to the description of fluctuations around the thermodynamic equilibrium state (as Mori-Zwanzig's derivation is).

This more powerful perspective is rooted in the general and fundamental laws of linear irreversible thermodynamics and the corresponding stochastic theory of fluctuations, as stated by Onsager [31, 32] and by Onsager and Machlup [33, 34], respectively, with an adequate extension [27, 28] to include non-Markov processes, which allows the description of relaxation phenomena that involve memory effects. The fact that it is based on mathematical symmetries explains the most important feature of Onsager's phenomenological line of reasoning, namely, its remarkable flexibility.

Thus, since the mathematical condition of stationarity [27], and not the physical condition of thermodynamic equilibrium, is the essence of Onsager's formalism (including the GLE in Eq. (SM1)), there is no fundamental impediment to consider its application to the description of non-equilibrium stationary states [35]. Furthermore, modeling a *non-stationary* stochastic process as a piecewise sequence of stationary processes, allows us to build a non-stationary version of the GLE [36]. As a result, one can envision a general route to the description of non-equilibrium and non-stationary processes [37]. It was precisely along this route, applied to the context of simple liquids, that such non-stationary canonical and abstract GLE formalism became a generic first-principles non-equilibrium theory of the structure and dynamics of liquids.

General NE-SCGLE theory.

Ref. [1] explains in detail how this non-stationary extension of the GLE formalism resulted in what we refer to as the *non-equilibrium self-consistent generalized Langevin equation* (NE-SCGLE) theory of irreversible processes in liquids. The central elements of this generic theory are the time evolution equations describing the kinetics of the mean value $\overline{n}(\mathbf{r},t)$ and of the covariance $\sigma(\mathbf{r},\mathbf{r}';t) \equiv \overline{\delta n(\mathbf{r},t)\delta n(\mathbf{r}',t)}$, of the fluctuations $\delta n(\mathbf{r},t) = n(\mathbf{r},t) - \overline{n}(\mathbf{r},t)$ of the instantaneous local density $n(\mathbf{r},t)$. The first of these equations reads [1]

$$\frac{\partial \overline{n}(\mathbf{r},t)}{\partial t} = D^0 \nabla \cdot b(\mathbf{r},t)\overline{n}(\mathbf{r},t)\nabla\beta\mu[\mathbf{r};\overline{n}(t)], \qquad \text{(SM2)}$$

where $\mu[\mathbf{r};n]$ is the electrochemical potential at position $\mathbf{r}$ (which is a *functional* of the local concentration profile $n(\mathbf{r})$ [38]). This is just Fick's law in its most general form, which involves the dimensionless local mobility function $b(\mathbf{r},t)$ that describes the local frictional effects of the direct interactions between particles. If we set $b(\mathbf{r},t) = 1$ in this equation, it becomes the fundamental equation of dynamic density functional theory [39].

As explained in Ref. [1], the time-evolution equation for the mean value $\overline{n}(\mathbf{r},t)$ in Eq. (SM2) is intimately related with the time-evolution equation for the covariance $\sigma(\mathbf{r},\mathbf{r}';t)$, which then reads

$$\begin{aligned} \frac{\partial \sigma(\mathbf{r},\mathbf{r}';t)}{\partial t} &= \\ D^0\nabla\cdot\overline{n}(\mathbf{r},t)\; b(\mathbf{r},t)\nabla &\int d\mathbf{r}_1 \mathcal{E}[\mathbf{r},\mathbf{r}_1;\overline{n}(t)]\sigma(\mathbf{r}_1,\mathbf{r}';t) \\ + D^0\nabla'\cdot\overline{n}(\mathbf{r}',t)\; b(\mathbf{r}',t)\nabla' &\int d\mathbf{r}_1 \mathcal{E}[\mathbf{r}',\mathbf{r}_1;\overline{n}(t)]\sigma(\mathbf{r}_1,\mathbf{r};t) \\ - 2D^0\nabla\cdot\overline{n}(\mathbf{r},t)\; b(\mathbf{r},t)&\nabla\delta(\mathbf{r}-\mathbf{r}'). \end{aligned} \qquad \text{(SM3)}$$

with $\mathcal{E}[\mathbf{r},\mathbf{r}';\overline{n}(t)]$ being the functional derivative $\mathcal{E}[\mathbf{r},\mathbf{r}';n] \equiv [\delta\beta\mu[\mathbf{r};n]/\delta n(\mathbf{r}')]$ evaluated at $\overline{n}(\mathbf{r},t)$.

For given D_0 and $\mu[\mathbf{r};n]$, Eqs. (SM2) and (SM3) would constitute a closed system of equations for $\overline{n}(\mathbf{r},t)$ and $\sigma(\mathbf{r},\mathbf{r}';t)$. This, however, is not the case because in reality the mobility function $b(\mathbf{r},t)$ happens to be a state function, i.e., it is also a *functional* of $\overline{n}(\mathbf{r},t)$ and $\sigma(\mathbf{r},\mathbf{r}';t)$. The corresponding *exact* functional dependence is in general unknown. However, providing an approximate but general proposal for this dependence is precisely the essence of the NE-SCGLE theory. The most general version of such proposal is summarized by a set of equations that write $b(\mathbf{r},t)$ in terms not only of $\overline{n}(\mathbf{r},t)$ and $\sigma(\mathbf{r},\mathbf{r}';t)$, but also of the two-time correlation (or van Hove) function $C(\mathbf{r},\mathbf{r}';t,t') \equiv \overline{\delta n(\mathbf{r},t)\delta n(\mathbf{r}',t')}$. These are Eqs. (4.4)-(4.7) of Ref. [1], which we reproduce below (Eqs. (SM7)-(SM10)) after introducing two simplifying approximations.

To explain the first of them, let us write the position $\mathbf{r}'$ and the time t' as $\mathbf{r}' = \mathbf{r}+\mathbf{x}$ and $t' = t+\tau$, so that the van Hove function and the covariance can be written, respectively, as $C(\mathbf{x},\tau;\mathbf{r},t) \equiv \overline{\delta n(\mathbf{r},t)\delta n(\mathbf{r}+\mathbf{x};t+\tau)}$ and $\sigma(\mathbf{x};\mathbf{r},t) \equiv \overline{\delta n(\mathbf{r},t)\delta n(\mathbf{r}+\mathbf{x};t)}$. Clearly, these two properties depend in general on the location $\mathbf{r}$ in space and on the direction of the relative-position vector $\mathbf{x}$, i.e., they are not homogeneous nor isotropic. Let us, however, assume for simplicity that these functions are locally isotropic, i.e., that within a small volume around the position $\mathbf{r}$, they only depend on the magnitude $|\mathbf{x}|$ of the relative position vector $\mathbf{x}$. Under these conditions, for example, the local covariance $\sigma(|\mathbf{x}|;\mathbf{r},t)$ can be written in terms of its Fourier transform $\sigma(k;\mathbf{r},t)$ as

$$\sigma(|\,\mathbf{x}\,|;\mathbf{r},t) = \frac{1}{(2\pi)^3}\int d^3k e^{-i\mathbf{k}\cdot\mathbf{x}}\sigma(k;\mathbf{r},t). \qquad \text{(SM4)}$$

This allows us to rewrite Eq. (SM3) as

$$\begin{aligned} \frac{\partial\sigma(k;\mathbf{r},t)}{\partial t} =& -2k^2D^0\overline{n}(\mathbf{r},t)b(\mathbf{r},t)\mathcal{E}(k;\overline{n}(\mathbf{r},t))\sigma(k;\mathbf{r},t) \\ &+ 2k^2D^0\overline{n}(\mathbf{r},t)\; b(\mathbf{r},t), \end{aligned} \qquad \text{(SM5)}$$

where $\mathcal{E}(k;\overline{n}(\mathbf{r},t)) \equiv (2\pi)^{-3}\int d^3k e^{-i\mathbf{k}\cdot\mathbf{x}}\mathcal{E}[\mathbf{r},\mathbf{r}+\mathbf{x};\overline{n}(\mathbf{r},t)]$.

Spatially-uniform NE-SCGLE theory.

After introducing the previous approximation of local isotropy, we can say that Eqs. (SM2) and (SM5)

above, together with Eqs. (4.4)-(4.7) of Ref. [1], are thus the essence of the NE-SCGLE theory in its most general version. The actual solution of these equations, however, may involve a considerable mathematical and numerical effort. In the absence of external fields, however, we may devise a simple strategy to have a glimpse of their most relevant predictions. Such a strategy constitutes our second simplifying approximation, and starts by writing $\overline{n}(\mathbf{r},t)$, $b(\mathbf{r},t)$, and $\sigma(k;\mathbf{r},t)$ as the sum of their homogeneous mean (or bulk) values $\overline{n}$, $b(t)$, and $\sigma(k;t)$, plus their deviations from homogeneity, $\Delta\overline{n}(\mathbf{r},t)$ $(\equiv \overline{n}(\mathbf{r},t) - \overline{n})$, $\Delta b(\mathbf{r},t)$ $(\equiv b(\mathbf{r},t) - b(t))$, and $\Delta\sigma(k;\mathbf{r},t)$ $(\equiv \sigma(k;\mathbf{r},t) - \sigma(k;t))$. We then start by neglecting these deviations, so that Eq. (SM2) becomes irrelevant, and Eq. (SM5) for $\sigma(k;\mathbf{r},t)$, now becomes the time-evolution equation for the non-equilibrium structure factor $S(k;t) \equiv \sigma(k;t)/\overline{n}$ of the instantaneously-quenched liquid at waiting time $t > 0$ after the quench. Denoting from now on this waiting time t as t_w, such an equation reads

$$\frac{\partial S(k;t_w)}{\partial t_w} = -2k^2 D^0 b(t_w) n\mathcal{E}_f(k)\left[S(k;t_w) - 1/n\mathcal{E}_f(k)\right], \tag{SM6}$$

where $\mathcal{E}_f(k)$ is the Fourier transform (FT) of the functional derivative $\mathcal{E}[\mid \mathbf{r}-\mathbf{r}' \mid; n, T] \equiv [\delta\beta\mu[\mathbf{r}; n, T]/\delta n(\mathbf{r}')]$.

This equation is then complemented with the homogeneous version of the expression for $b(\mathbf{r},t)$ in Eq. (4.4) of Ref. [1], namely,

$$b(t_w) = [1 + \int_0^\infty d\tau \Delta\zeta^*(\tau;t_w)]^{-1}, \tag{SM7}$$

for the t_w-dependent mobility $b(t_w)$ in terms of the t_w-evolving, τ-dependent friction function $\Delta\zeta^*(\tau;t_w)$, given by the simplified version of Eq. (4.7) of Ref. [1], namely,

$$\Delta\zeta^*(\tau;t_w) = \frac{D_0}{24\pi^3 n}\int d\mathbf{k}\; k^2\left[\frac{S(k;t_w)-1}{S(k;t_w)}\right]^2 \times F(k,\tau;t_w)F_S(k,\tau;t_w) \tag{SM8}$$

in terms of $S(k;t_w)$ and of the collective and self non-equilibrium intermediate scattering functions $F(k,\tau;t_w)$ and $F_S(k,z;t_w)$, whose memory-function equations are written approximately, in terms of the Laplace transforms (LT) $F(k,z;t_w)$ and $F_S(k,\tau;t_w)$, as

$$F(k,z;t_w) = \frac{S(k;t_w)}{z + \frac{k^2 D^0 S^{-1}(k;t_w)}{1+\lambda(k)\;\Delta\zeta^*(z;t_w)}}, \tag{SM9}$$

and

$$F_S(k,z;t_w) = \frac{1}{z + \frac{k^2 D^0}{1+\lambda(k)\;\Delta\zeta^*(z;t_w)}}, \tag{SM10}$$

where

$$\lambda(k) \equiv 1/[1 + (k/k_c)^2] \tag{SM11}$$

is an "interpolating function" [17], with k_c being an empirically determined parameter. In the present work we use $k_c = 1.305(2\pi)/\sigma$, with σ being the hard-core diameter [40].

Solving Eqs. (SM6)-(SM11) yields the full non-equilibrium evolution (i.e., the t_w-dependence) of all the properties explicitly involved in these equations, namely, $S(k;t_w)$, $b(t_w)$, $\Delta\zeta^*(\tau;t_w)$, $F(k,\tau;t_w)$, and $F_S(k,z;t_w)$, from which still other non-equilibrium properties may be derived. For example, the equilibration or aging of the system may also be monitored through its t_w-dependent mean squared displacement (MSD), defined as $W(\tau;t_w) \equiv < [\mathbf{R}(t_w+\tau) - \mathbf{R}(t_w)]^2 > /6$. This dynamic property may be determined as the solution of

$$W(\tau;t_w) = D^0\tau - \int_0^\tau \Delta\zeta^*(\tau-\tau';t_w)W(\tau';t_w)d\tau'. \tag{SM12}$$

Let us finally notice that in the equilibrium limit, $S(k;t_w \to \infty) = S^{eq}(k)$, Eqs. (SM8)-(SM11) become the equilibrium version of the SCGLE theory. The resulting equations are quite similar but somewhat simpler than the corresponding MCT equations. In particular, although the expression for the equilibrium time-dependent tracer friction function $\Delta\zeta^{*eq}(\tau) \equiv \Delta\zeta^*(\tau;t_w\to\infty)$ of both theories are identical, MCT and SCGLE follow different strategies to approximate the collective and self memory functions. This leads to differences in some details, whose consequences have recently been discussed in Ref. [22].

Brief review of applications.

The self-consistent system of equations above, Eqs. (SM6)-(SM11), summarizes the simplest, spatially homogeneous, version of the NE-SCGLE theory to zeroth order in $\Delta\overline{n}(\mathbf{r},t)$, employed in this and in previous studies [2, 3]. This theory involves only spatially uniform properties, such as the non-equilibrium structure factor $S(k;t_w)$ and the uniform but t_w-dependent local mobility function $b(t_w)$. Until now, only this zeroth-order version of the NE-SCGLE theory has been quantitatively applied, exhibiting an amazing predictive power and ability to explain many of the fundamental features of the glass and the gel transition.

For example, for simple liquids with purely repulsive interactions, the solution of the NE-SCGLE above provides a detailed description of the non-stationary and non-equilibrium transformation of equilibrium hard- (and soft)-sphere liquids, into "repulsive" (high-temperature, high-density) hard-sphere glasses [41]. These predictions have recently found a reasonable agreement with the corresponding non-equilibrium simulations [42], naturally explaining some of the most essential signatures of the glass transition [43–45].

When extended to simple liquids that also include an attractive interaction (which we refer to as "Lennard-Jones(LJ)–like" liquids), the analysis of the stationary solutions of the NE-SCGLE non-linear equations above (Eqs. (SM6)-(SM11)) predicts a much richer and detailed long-time asymptotic scenario involving new dynamically-arrested phases [2], identified with the formation of gels and porous glasses [46–54].

However, in stark contrast with the approach to thermodynamic equilibrium, whose long-time asymptotic scenario is established within *finite* equilibration times [40, 42], reaching dynamically arrested non-equilibrium states is predicted [2, 41, 42] to involve increasingly slower and imperceptible aging processes. This then implies that the relevant properties of the non-equilibrium arrested states observed in any real experiment, are in reality non-stationary and non-equilibrium in nature. Hence, they can only be understood from a kinetic perspective, such as that provided by the NE-SCGLE theory itself. Precisely this use of the NE-SCGLE theory was recently illustrated [3] with the demonstration that its predicted full time-evolution of the structure factor $S(k; t_w)$ of a simple LJ–like liquid after being instantaneously quenched to the interior of its spinodal region, exhibits the main structural fingerprints of arrested spinodal decomposition associated with the formation of dynamically arrested spongelike amorphous materials [46–54].

The NE-SCGLE theory was recently extended to multi-component systems [55], thus opening the possibility of studying the non-equilibrium aging processes in liquid mixtures. For example, one can now describe the aging processes occurring in binary hard-sphere mixtures during the formation of the predicted [56, 57] "double glasses" (with the simultaneous arrest of both species) and single glasses (formed by the arrested large particles with the small species remaining mobile), recently confirmed experimentally and by simulations [58].

Let us finally mention that the NE-SCGLE theory has also been extended to liquids formed by particles interacting by non-radially symmetric forces [59, 60], thus opening the route to the description of even more subtle and complex non-equilibrium amorphous states of matter.

LATENCY TIMES AND RHEOMETRY EXPERIMENTS.

In this section we discuss the details of the comparison of our theoretical predictions for the latency time, with the experimental data of Fig. 7(a) of Ref. [61], presented in the inset of Fig. 1(b) of the Letter. That reference reports the rheometry measurements of the temporal evolution of gel formation in suspensions of silica colloids with tunable short-range attractions. After a sudden quench, this system displays a distinct latency period in which the suspension remains fluid before exhibiting measurable elastic properties. This experimental latency time t_G quantifies the duration of these fluid properties preceding the onset of solid-like response.

To compare our theoretical results with the experimental data of Ref. [61], we took the diameter of the particles declared in the experiments, namely, $\sigma = 44$ nm, and the short time self-diffusion coefficient as 2.90×10^{-12} m^2/s. This value was obtained via the Stokes-Einstein relation, using the same particle diameter and the viscosity of the solvent $\eta = 3.355$ mPa·s, for the temperature T=293.15 K. We used $t_0 \equiv \sigma^2/D_0 = 6.65 \times 10^{-4}$s to scale the experimental time. In both cases, experimental and theoretical data corresponds to a volume fraction of $\phi = 0.20$. Using this information, we can plot a dimensionless latency time for different quenches as a function of the scaled temperature $T^* = (T - T_s)/T_s$, shown in the inset of Figure 1(b) of our Letter, where T_s is the spinodal temperature.

We can observe that the experimental and theoretical latency times have the same asymptotic functional dependence (proportional to $T^{*\alpha}$, with $\alpha \approx 2.5$). The quantitative difference in the proportionality prefactor could be due to the difference between the experimental system and the theoretical model. The comparison between them has the purpose of exhibiting the essential features of the dynamic properties of the spinodal decomposition process obtained by the theoretical approach and the experimental results.

TWO-STEP RELAXATION EXHIBITED BY THE SELF ISF $F_S(k, \tau; t_w)$.

This section presents the non-equilibrium kinetics of the self-intermediate scattering function $F_S(k, \tau; t_w)$. For simplicity, we basically use the same format employed to illustrate the non-equilibrium kinetics of the mean square displacement $W(\tau; t_w, T_f)$ in Fig. 2 of the Letter (main panels). Thus, in Fig. 1 here, we plot $F_S(k = 1, \tau; t_w, T_f)$ as a function of correlation time τ for the same sequence of waiting times t_w and the same two representative instantaneous quenches discussed there. These two quenches correspond to final temperatures $T_f^{(3)} = 0.18$ and $T_f^{(2)} = 0.23$, whose corresponding NE-SCGLE results for $F_S(k = 1, \tau; t_w, T_f)$ are presented, respectively, in Figs. 1(a) and 1(b).

In Fig. 1(a) we illustrate the relaxation of $F_S(k = 1, \tau; t_w, T_f)$ after a deep quench, to a final temperature slightly *below* the gel-glass transition, $T_f < T_c(\phi)$. This is to be compared with the results in Fig. 1(b) for the relaxation of $F_S(k = 1, \tau; t_w, T_f)$ after a similarly deep quench, but whose final temperature lies only slightly *above* this transition. Let us first notice that in quench 3, $F_S(k = 1, \tau; t_w, T_f)$ first decays to a plateau

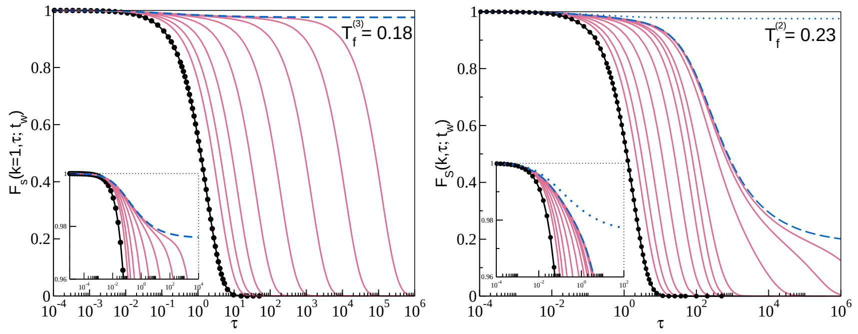

FIG. SM 1: NE-SCGLE theoretical snapshots of the non-equilibrium self-intermediate scattering function $F_S(k=1,\tau;t_w,T_f)$, plotted as a function of correlation time τ for waiting times $t_w=0$ (line with dark circles), $t_w=10^{0.5n}$, with $n=-3,-2,-1,\cdots$ (solid lines), and $t_w=\infty$ (dashed lines), after a quench to final temperature: (a) $T_f^{(3)}=0.18$ and (b) $T_f^{(2)}=0.23$.

(β-relaxation), determined by its non-ergodicity parameter $f_S(k;T_f)\equiv\lim_{\tau;t_w\to\infty}F_S(k,\tau;t_w,T_f)$ (dotted line), to finally decay to zero as $\tau\to\infty$ (α-relaxation). This relaxation pattern is identical to the aging of dense soft-sphere glass-forming liquids (see, for example, Fig. 12(a) of Ref. [41] and Fig. 1(b) of Ref. [42], which involve, however, a larger wave-vector, $k=7.1$).

In comparison, we see that in quench 2 for waiting times shorter than about 10^3, $F_S(k=1,\tau;t_w,T_f)$ exhibits a similar (although faster) relaxation pattern, first decaying to the same plateau (β-relaxation), followed by the final (α-relaxation). For waiting times longer than 10^3, however, a second plateau emerges at longer τ (dashed line of Fig. 1(b)), determined by the non-ergodicity parameter $f_S(k;T_f^{(2)})$ corresponding to quench 2 (dashed line). This two-step structural relaxation is clearly the most apparent effect in $F_S(k,\tau;t_w,T_f)$ of the competition between the porous glass transition ($T_c=0.22$) and gelation [62–64].

DETERMINATION OF THE CROSSOVER TEMPERATURE $T_0(\phi)$

In this section we provide additional information on the procedure performed to determine the crossover temperature $T_0(\phi)$, which divides the interval $T_c(\phi)\le T_f\le T_s(\phi)$ of the dynamic arrest diagram, in two well-defined sub-regimes: the subinterval $T_c(\phi)\le T_f\le T_0(\phi)$, where $d_l(T_f)$ increases exponentially with T_f (Region I), and the subinterval $T_0(\phi)\le T_f\le T_s(\phi)$, where $d_l(T_f)$ enters its diverging power-law regime (Region II).

For this, let us first recall that the results illustrated in Fig. 3(a) of our manuscript for the long-time asymptotic value of the squared localization length, $\lambda^2(T_f)=\gamma_a(T_f)\equiv W_a(\tau\to\infty,T_f)$ could be conveniently parametrized in Ref. [2] with an empiric expression that matches an exponential function below T_0 with a power law above T_0, in a manner that both, the function and its derivative, are continuous at T_0. This procedure led to the determination of $T_0(\phi)$ reported in Fig. 10 of Ref. [2], reproduced as the empty circles of Fig. 3(b) of our present manuscript.

We have implemented exactly the same analysis of our results for the latency distance $d_l(T_f)$. Thus, in Fig. SM2 we reproduce the results for $d_l(T_f)$ in Fig. 3(a) of the manuscript, to illustrate the fact that $d_l(T_f)$ exhibits two sub-regimes, characterized by a different dependence on the final temperature. In the first sub-regime $d_l(T_f)$ increases exponentially with T_f, whereas in the second $d_l(T_f)$ increases with T_f as an inverse power that diverges at the spinodal line. Thus, following Ref. [2], we propose the following parametrization of the T_f-dependence of $d_l(T_f)$,

$$\begin{aligned} d_l(T_f) &= d_l(T_c)e^{\alpha\left(\frac{T_f-T_c}{T_s-T_0}\right)}, \quad \text{for,} \quad T_c\le T_f\le T_0 \\ &= d_l^*(T_c)e^{\alpha\left(\frac{T_0-T_c}{T_s-T_0}\right)}\left[\frac{T_s-T_f}{T_s-T_0}\right]^{-\alpha}, \text{for,} \quad T_0\le T_f\le T_s. \end{aligned} \tag{SM13}$$

We found that a reasonable representation of the numerical results for $d_l(T_f)$ in Fig. 3(a) could be obtained with the exponent α fixed as $\alpha=1.25$, and adjusting T_0 to find the best overall fit, illustrated in Fig. SM2 for the isochore $\phi=0.2$, leading to the value $T_0(\phi=0.2)=0.51$. We tested the same procedure for other isochores, also fixing the exponent α as $\alpha=1.25$, and determined $T_0(\phi)$ from the best overall fit. This procedure provides the second determination of the location of the crossover curve $T_0(\phi)$, represented by the solid circles of Fig. 3(b) of our manuscript.

[1] P. E. Ramírez-González and M. Medina-Noyola, Phys. Rev. E **82**, 061503 (2010).
[2] J. M. Olais-Govea, L. López-Flores, and M. Medina-Noyola, J. Chem Phys. **143**, 174505 (2015).

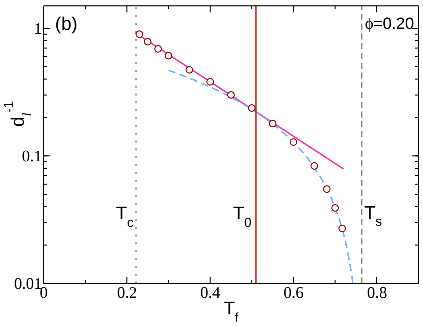

FIG. SM 2: Numerical results for the NE-SCGLE inverse latency distance $d_l^{-1}(T_f)$ (solid circles) as a function of T_f, along the isochore $\phi = 0.2$. The solid line is the exponential fit of the data for $T_c \le T_f \le T_0$ whereas the dashed line is the power-law fit for $T_0 \le T_f \le T_s$, according to Eq. (SM13) with $\alpha = 1.25$ and $T_0 = 0.51$.

[3] J. M. Olais-Govea, L. López-Flores, and M. Medina-Noyola, Phys. Rev. E **98**, 040601(R) (2018).
[4] W. Götze and E. Leutheusser, Phys. Rev. A **11**, 2173 (1975).
[5] L Sjogren and A Sjolander, J. Phys. C: Solid State Phys. **12**, 4369 (1979).
[6] W. Götze and L. Sjögren, Rep. Prog. Phys. **55**, 241 (1992).
[7] W. Götze, *Complex Dynamics of Glass-Forming Liquids: A Mode-Coupling Theory*, Oxford University Press (2009).
[8] W. van Megen and P. N. Pusey, Phys. Rev. A **43**, 5429 (1991).
[9] F. Sciortino and P. Tartaglia, Adv. Phys. **54**, 471 (2005).
[10] L. Yeomans-Reyna and M. Medina-Noyola, Phys. Rev. E **62**, 3382 (2000).
[11] L. Yeomans-Reyna and M. Medina-Noyola, Phys. Rev. E **64**, 066114 (2001).
[12] L. Yeomans-Reyna, H. Acuña-Campa, F. Guevara-Rodríguez, and M. Medina-Noyola, Phys. Rev. E **67**, 021108 (2003).
[13] M. A. Chávez-Rojo and M. Medina-Noyola, Physica A **366**, 55 (2006).
[14] M. A. Chávez-Rojo and M. Medina-Noyola, Phys. Rev. E **72**, 031107 (2005); ibid **76**: 039902 (2007).
[15] P.E. Ramírez-González *et al.*, Rev. Mex. Física **53**, 327 (2007).
[16] L. Yeomans-Reyna *et al.*, Phys. Rev. E **76**, 041504 (2007).
[17] R. Juárez-Maldonado *et al.*, Phys. Rev. E **76**, 062502 (2007).
[18] P. E. Ramírez-González *et al.*, J. Phys.: Cond. Matter, **20**: 20510 (2008).
[19] R. Juárez-Maldonado and M. Medina-Noyola, Phys. Rev. E **77**, 051503 (2008).
[20] R. Juárez-Maldonado and M. Medina-Noyola, Phys. Rev. Lett. **101**, 267801 (2008).
[21] P. E. Ramírez-González and M. Medina-Noyola, J. Phys.: Cond. Matter, **21**, 75101 (2009).
[22] L. F. Elizondo-Aguilera and Th. Voigtmann, Phys. Rev. E **100**, 042601 (2019).
[23] R. Zwanzig, Phys. Rev. **124**, 983 (1961).
[24] H. Mori, Prog. Theor. Phys. **33**, 423 (1965).
[25] R. Kubo, Rep. Prog. Phys., **29** 255 (1966).
[26] B. Berne, "Projection Operator Techniques in the theory of fluctuations", in *Statistical Mechanics, Part B: Time-dependent Processes*, B. Berne, ed. (Plenum, New York, 1977).
[27] M. Medina-Noyola and J. L. del Río-Correa, *Physica 146 A*, 483 (1987).
[28] M. Medina-Noyola, *Faraday Discuss. Chem. Soc.* **83**, 21 (1987).
[29] J.R.D. Copley and S.W. Lovesey, Rep. Prog. Phys., **38**, 461 (1975).
[30] J. L. Boon and S. Yip, *Molecular Hydrodynamics* (Dover Publications Inc. N. Y., 1980).
[31] L. Onsager, Phys. Rev. **37**, 405 (1931).
[32] L. Onsager, Phys. Rev. **38**, 2265 (1931).
[33] L. Onsager and S. Machlup, Phys. Rev. **91**, 1505 (1953).
[34] S. Machlup and L. Onsager, Phys. Rev. **91**, 1512 (1953).
[35] F. Donado, R. E. Moctezuma, L. López-Flores, M. Medina-Noyola and J. L. Arauz-Lara, Scientific Reports, 7, 12614 (2017).
[36] M. Medina-Noyola, arXiv:0908.0521v1 [cond-mat.stat-mech] (2009).
[37] P. E. Ramírez-González and M. Medina-Noyola, J. Phys.: Cond. Matter **21**: 504103 (2009).
[38] R. Evans, Adv. Phys. **28**: 143(1979).
[39] U. Marini Bettolo Marconi and P. Tarazona, J. Chem. Phys. **110**, 8032 (1999); ibid., J. Phys.: Condens. Matter **12**, A413 (2000)
[40] G. Perez-Ángel et al., Phys. Rev. E **83**, 060501(R) (2011).
[41] L. E. Sánchez-Díaz, P. E. Ramírez-González, and M. Medina-Noyola, Phys. Rev. E **87**, 052306 (2013).
[42] P. Mendoza-Méndez, E. Lázaro-Lázaro, L. E. Sánchez-Díaz, P. E. Ramírez-González, G. Pérez-Ángel, and M. Medina-Noyola, Phys. Rev. E **96**, 022608 (2017).
[43] Angell C. A., Ngai K. L., McKenna G. B., McMillan P. F. and Martin S. F., J. Appl. Phys. **88** 3113 (2000).
[44] M. D. Ediger, C. A. Angell, and S. R. Nagel, J. Phys. Chem. **100**, 13200 (1996).
[45] K. L. Ngai, D. Prevosto, S. Capaccioli and C. M. Roland, J. Phys.: Condens. Matter **20**, 244125 (2008).
[46] P. J. Lu, E. Zaccarelli, F. Ciulla, A. B. Schofield, F. Sciortino and D. Weitz, Nature **453**, 499 (2008).
[47] E. Sanz, M. E. Leunissen, A. Fortini, A. van Blaaderen, and M. Dijkstra, J. Phys. Chem. B **112**, 10861 (2008).
[48] T. Gibaud and P. Schurtenberger, J. Phys.: Condens. Matter **21**, 322201 (2009).
[49] L. Di Michele, D. Fiocco, F. Varrato, S. Sastry, E. Eisera and G. Foffi, Soft Matter **10**, 3633 (2014).
[50] Y. Gao, J. Kim, and M. E. Helgeson, Soft Matter, **11**, 6360-6370 (2015).
[51] S. Da Vela et al., Soft Matter, **12**, 9334 (2016).
[52] I. Zhang, C. P. Royall, M. A. Faersd and P. Bartlett, Soft Matter, **9**, 2076, (2013).
[53] J. F. M. Lodge and D. M. Heyes, J. Chem. Soc., Faraday Trans., **93**, 437 (1997).
[54] V. Testard, L. Berthier, and W. Kob, J. Chem. Phys. **140**, 164502 (2014).
[55] L. E. Sánchez-Díaz, E. Lázaro-Lázaro, J. M. Olais-Govea and M. Medina-Noyola, J. Chem Phys. **140**, 234501 (2014).
[56] Th. Voigtmann, Europhys. Lett. 96, 36006 (2011).
[57] R. Juárez-Maldonado and M. Medina-Noyola, Phys.Rev. E, 77, 051503 (2008); Ibid Phys.Rev. Lett., 101, 267801

(2008).
[58] E. Lázaro-Lázaro et al., Phys. Rev. E 99, 042603 (2019).
[59] L.F. Elizondo-Aguilera, P. F. Zubieta-Rico, H. Ruíz Estrada, and O. Alarcón-Waess, Phys. Rev. E, **90**, 052301 (2014).
[60] E. Cortés-Morales, L.F. Elizondo-Aguilera, and M. Medina-Noyola, J. Phys. Chem. B, 120 (32), pp 7975-7987 (2016).
[61] H. Guo, S. Ramakrishnan, J. L. Harden, and R. L. Leheny, J. Chem. Phys. **135**, 154903 (2011).
[62] N. Khalil, A. de Candia, A. Fierro, M. P. Cimarra and A. Coniglio, Soft Matter, **10**, 4800 (2014).
[63] P. Chaudhuri, L. Berthier, P. I. Hurtado, and W. Kob, Phys. Rev. E **81**, 040502(R) (2010).
[64] P. Chauduri, P. I. Hurtado, L. Berthier and W. Kob, J. Chem Phys. **142**, 174503 (2015).
